# 2-[1-(2-Hydroxy-3-methoxybenzyl)-1*H*-benzimidazol-2-yl]-6-methoxyphenol monohydrate

**DOI:** 10.1107/S1600536809010769

**Published:** 2009-03-28

**Authors:** Mohammed H. Al-Douh, Hasnah Osman, Shafida A. Hamid, Reza Kia, Hoong-Kun Fun

**Affiliations:** a School of Chemical Sciences, Universiti Sains Malaysia, 11800 USM, Penang, Malaysia; b Kulliyyah of Science, International Islamic University Malaysia (IIUM), Jalan Istana, Bandar Indera Mahkota 25200 Kuantan, Pahang, Malaysia; c X-ray Crystallography Unit, School of Physics, Universiti Sains Malaysia, 11800 USM, Penang, Malaysia

## Abstract

The asymmetric unit of the title compound, C_22_H_20_N_2_O_4_·H_2_O, comprises a substituted benzimidazole molecule and a water mol­ecule of crystallization. The dihedral angles between the benzimidazole ring system and the two outer benzene rings are 16.54 (4) and 86.13 (4)°. The dihedral angle between the two hydr­oxy-substituted benzene rings is 82.20 (5)°. In the crystal structure, inter­molecular O—H⋯O hydrogen bonds, involving the hydr­oxy groups and water mol­ecules, form *R*_4_^4^(8) ring motifs, and link symmetry-related mol­ecules into extended chains along the *c* axis. The crystal structure is further stabilized by weak inter­molecular C—H⋯O hydrogen bonds, weak C—H⋯π and π–π stacking [centroid–centroid = 3.6495 (6)–3.7130 (6) Å] inter­actions. Intra­molecular O—H⋯O and O—H⋯N inter­actions are also present.

## Related literature

For hydrogen-bond motifs, see Bernstein *et al.* (1995 ▶). For the synthesis and bioactivity of benzimidazoles see, for example: Soto *et al.* (2006 ▶); Vazquez *et al.* (2006 ▶); Latif *et al.* (1983 ▶). For related structures, see: Elerman & Kabak (1997 ▶); Liu *et al.* (2006 ▶); Al-Douh *et al.* (2006 ▶, 2007 ▶). For the stability of the temperature controller used for the data collection, see: Cosier & Glazer (1986 ▶).
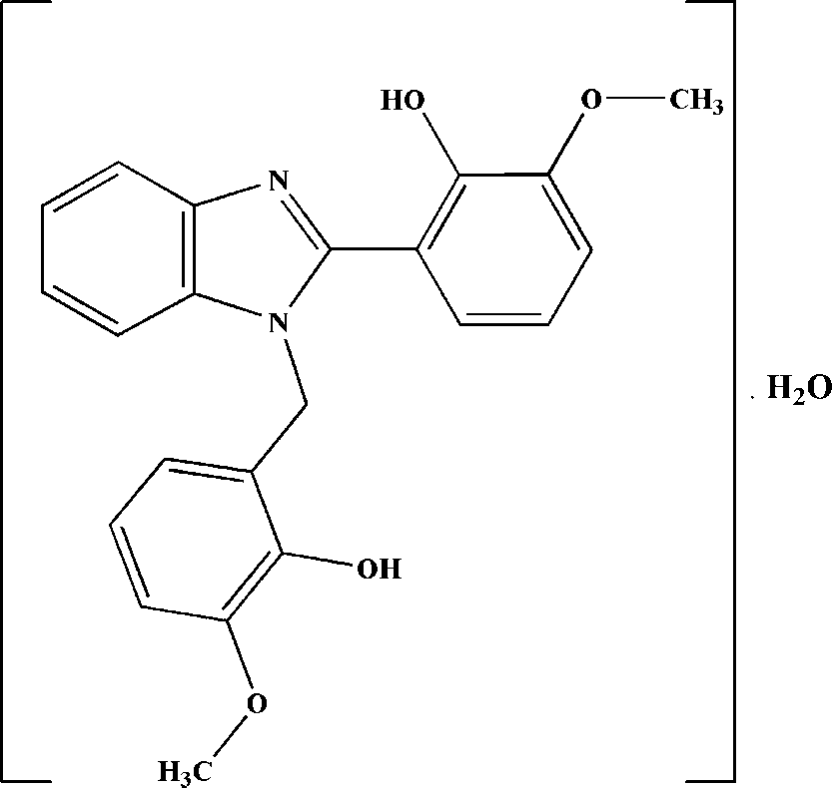


## Experimental

### 

#### Crystal data

C_22_H_20_N_2_O_4_·H_2_O
*M* *_r_* = 394.42
Triclinic, 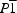

*a* = 7.5076 (1) Å
*b* = 9.8557 (1) Å
*c* = 13.2240 (2) Å
α = 106.306 (1)°
β = 97.135 (1)°
γ = 97.993 (1)°
*V* = 916.18 (2) Å^3^
*Z* = 2
Mo *K*α radiation
μ = 0.10 mm^−1^
*T* = 100 K
0.48 × 0.28 × 0.10 mm

#### Data collection

Bruker SMART APEXII CCD area-detector diffractometer
Absorption correction: multi-scan (**SADABS**; Bruker, 2005 ▶) *T*_min_ = 0.952, *T*_max_ = 0.990
33715 measured reflections
8009 independent reflections
6304 reflections with *I* > 2˘*I*)
*R*_int_ = 0.030

#### Refinement

*R*[*F* ^2^ > 2σ(*F* ^2^)] = 0.049
*wR*(*F* ^2^) = 0.133
*S* = 1.03
8009 reflections
274 parameters
H atoms treated by a mixture of independent and constrained refinement
Δρ_max_ = 0.58 e Å^−3^
Δρ_min_ = −0.33 e Å^−3^

### 

Data collection: *APEX2* (Bruker, 2005 ▶); cell refinement: *SAINT* (Bruker, 2005 ▶); data reduction: *SAINT*; program(s) used to solve structure: *SHELXTL* (Sheldrick, 2008 ▶); program(s) used to refine structure: *SHELXTL*; molecular graphics: *SHELXTL*; software used to prepare material for publication: *SHELXTL* and *PLATON* (Spek, 2009 ▶).

## Supplementary Material

Crystal structure: contains datablocks global, I. DOI: 10.1107/S1600536809010769/lh2793sup1.cif

Structure factors: contains datablocks I. DOI: 10.1107/S1600536809010769/lh2793Isup2.hkl

Additional supplementary materials:  crystallographic information; 3D view; checkCIF report

## Figures and Tables

**Table 1:** Hydrogen-bond geometry (Å, °)

*D*—H⋯*A*	*D*—H	H⋯*A*	*D*⋯*A*	*D*—H⋯*A*
O1—H1⋯N1	0.84	1.80	2.5447 (12)	147
O1*W*—H2*W*1⋯O1^i^	0.84 (2)	2.23 (2)	3.0151 (11)	155 (2)
O2—H2⋯O4	0.84	2.21	2.6650 (11)	114
O2—H2⋯O1*W*^ii^	0.84	1.95	2.7401 (11)	155
O1*W*—H1*W*1⋯O2^iii^	0.87 (2)	2.04 (2)	2.8987 (12)	168.5 (19)
C21—H21*B*⋯O1*W*^iv^	0.98	2.58	3.2762 (16)	128
C22—H22*A*⋯O3^v^	0.98	2.54	3.2071 (14)	126
C22—H22*B*⋯*Cg*1^vi^	0.98	2.80	3.5497 (13)	133

Symmetry codes: (i) 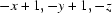; (ii) 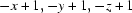; (iii) 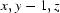; (iv) 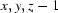; (v) 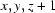; (vi) 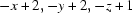. *Cg*1 is the centroid of the C15–C20 benzene ring.

**Fig. 1.**
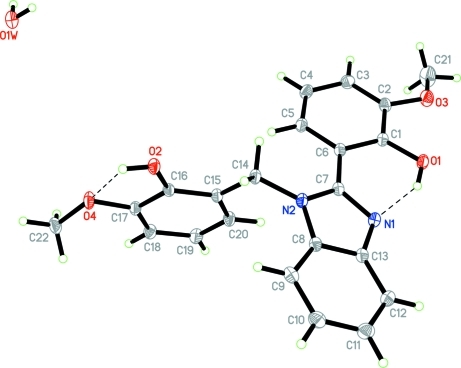
The molecular structure of the title compound, showing 50% probability displacement ellipsoids and the atomic numbering. Intramolecular H bonds are drawn as a dashed line.

**Fig. 2.**
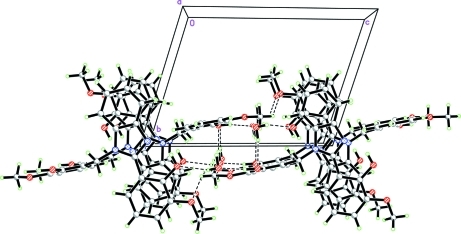
Part of the crystal structure of the title compound, viewed along the a-axis showing 1-D extended chains along the c-axis. Intermolecular interactions are drawn as dashed lines.

## supplementary crystallographic information

### Comment

Many benzimidazoles are pharmaceutical agents and are used widely in biological system applications which enable important synthetic strategies in drug discovery. Phenolic and anisolic benzimidazole derivatives have been synthesized and evaluated for vasodilator and antihypertensive activity (Soto *et al.*, 2006), while other alkyloxyaryl benzimidazole derivatives have been tested for the spasmolytic activity (Vazquez *et al.*, 2006). Latif *et al.* have developed the reactions of some phenolic aldehydes with *o*- phenylenediamine in great details and managed to isolate the title compound (Latif *et al.*, 1983). In view of the above, we have obtained the title compound (I), derived from benzimidazole and a bis-Schiff base compound and have determined its crystsal structure herein.

The bond lengths and angles in (I) are consistent with those common to related reported structures  (Elerman & Kabak, 1997; Liu *et al.*, 2006). The molecular structure of (I) is shown in Fig. 1. Intramolecular O—H···O and O—H···N hydrogen bonds generate five and six membered rings with *S(5)* and *S(6)* ring motifs respectively (Bernstein *et al.,* 1995). Intermolecular O—H···O hydrogen bonds, involving one of the hydroxy groups and one of the water molecules link neighbouring molecules into chains with *R^4^_4_(8)* ring motifs (Bernstein *et al.*, 1995). The dihedral angles between the benzimidazole ring system and the two outer benzene rings are 16.54 (4) and 86.13 (4)°. The dihedral angle between the two hydroxy substituted benzene rings is 82.20 (5)°. In the crystal structure the molecules are linked together by four-membered O—H···O—H···O—H interactions into 1-D extended chains along the *c* axis. The crystal structure is further stabilized by intermolecular C—H···O hydrogen bonds, weak intermolecular C—H···π (Table 1; *Cg*1 is the centroids of the C15–C20 benzene ring), and π-π interactions [*Cg*2···*Cg*2^vii^ = 3.6495 (6) Å; *Cg*3 ···*Cg*4^vii^ = 3.7130 (6) Å; *Cg*2, *Cg*3 and *Cg*4 are the centroids of the N1/C7/N2/C8/C13, C1–C6 and C8–C13 rings].

### Experimental

The synthetic method has been described earlier (Al-Douh *et al.*, 2006, 2007), while the single crystals suitable for X-ray diffraction were obtained by evaporation of a methanol solution of (I) at 353 K.

### Refinement

H atoms of the hydroxy groups were positioned by a freely rotating O—H bond and constrained with a fixed distance of 0.82 Å. The water H-atoms were located from the difference Fourier map and refined freely. The rest of the H atoms were positioned geometrically and refined with a riding model approximation with C—H = 0.95–0.98 and *U*_iso_(H) = 1.2 or 1.5 *U*_eq_(C). A rotating group model was used for the methyl groups.

### Crystal data

**Table:**

C_22_H_20_N_2_O_4_·H_2_O	*Z* = 2
*M**_r_* = 394.42	*F*(000) = 416
Triclinic, *P*1	*D*_x_ = 1.430 Mg m^−^^3^
Hall symbol: -P 1	Mo *K*α radiation, λ = 0.71073 Å
*a* = 7.5076 (1) Å	Cell parameters from 9908 reflections
*b* = 9.8557 (1) Å	θ = 2.5–33.4°
*c* = 13.2240 (2) Å	µ = 0.10 mm^−^^1^
α = 106.306 (1)°	*T* = 100 K
β = 97.135 (1)°	Plate, colourless
γ = 97.993 (1)°	0.48 × 0.28 × 0.10 mm
*V* = 916.18 (2) Å^3^	

### Data collection

**Table:**

Bruker SMART APEXII CCD area-detector diffractometer	8009 independent reflections
Radiation source: fine-focus sealed tube	6304 reflections with *I* > 2˘*I*)
graphite	*R*_int_ = 0.030
φ and ω scans	θ_max_ = 35.0°, θ_min_ = 2.2°
Absorption correction: multi-scan (*SADABS*; Bruker, 2005)	*h* = −10→12
*T*_min_ = 0.952, *T*_max_ = 0.990	*k* = −15→15
33715 measured reflections	*l* = −21→21

### Refinement

**Table:**

Refinement on *F*^2^	Primary atom site location: structure-invariant direct methods
Least-squares matrix: full	Secondary atom site location: difference Fourier map
*R*[*F*^2^ > 2σ(*F*^2^)] = 0.049	Hydrogen site location: inferred from neighbouring sites
*wR*(*F*^2^) = 0.133	H atoms treated by a mixture of independent and constrained refinement
*S* = 1.03	*w* = 1/[σ^2^(*F*_o_^2^) + (0.0658*P*)^2^ + 0.2927*P*] where *P* = (*F*_o_^2^ + 2*F*_c_^2^)/3
8009 reflections	(Δ/σ)_max_ = 0.001
274 parameters	Δρ_max_ = 0.58 e Å^−^^3^
0 restraints	Δρ_min_ = −0.33 e Å^−^^3^

### Special details

**Table:**

Experimental. The crystal was placed in the cold stream of an Oxford Cyrosystems Cobra open-flow nitrogen cryostat (Cosier & Glazer, 1986) operating at 100.0 (1) K.
Geometry. All e.s.d.'s (except the e.s.d. in the dihedral angle between two l.s. planes) are estimated using the full covariance matrix. The cell e.s.d.'s are taken into account individually in the estimation of e.s.d.'s in distances, angles and torsion angles; correlations between e.s.d.'s in cell parameters are only used when they are defined by crystal symmetry. An approximate (isotropic) treatment of cell e.s.d.'s is used for estimating e.s.d.'s involving l.s. planes.
Refinement. Refinement of *F*^2^ against ALL reflections. The weighted *R*-factor *wR* and goodness of fit *S* are based on *F*^2^, conventional *R*-factors *R* are based on *F*, with *F* set to zero for negative *F*^2^. The threshold expression of *F*^2^ > σ(*F*^2^) is used only for calculating *R*-factors(gt) *etc*. and is not relevant to the choice of reflections for refinement. *R*-factors based on *F*^2^ are statistically about twice as large as those based on *F*, and *R*- factors based on ALL data will be even larger.

### Fractional atomic coordinates and isotropic or equivalent isotropic displacement parameters (Å^2^)

**Table:**

	*x*	*y*	*z*	*U*_iso_*/*U*_eq_	
O1	0.70651 (10)	0.86574 (8)	−0.22860 (6)	0.01748 (14)	
H1	0.7410	0.9452	−0.1807	0.026*	
O2	0.57454 (9)	0.84985 (8)	0.36393 (6)	0.01561 (13)	
H2	0.5792	0.8439	0.4263	0.023*	
O3	0.65167 (12)	0.60902 (8)	−0.35747 (6)	0.02243 (16)	
O4	0.83510 (10)	0.78670 (9)	0.48995 (6)	0.01926 (15)	
N1	0.78166 (11)	1.04082 (9)	−0.04007 (7)	0.01479 (14)	
N2	0.68948 (10)	0.98309 (9)	0.09978 (6)	0.01414 (14)	
C1	0.68617 (12)	0.75865 (10)	−0.18267 (7)	0.01435 (16)	
C2	0.65860 (13)	0.61781 (11)	−0.25179 (8)	0.01672 (17)	
C3	0.63781 (14)	0.50113 (11)	−0.21256 (9)	0.01892 (18)	
H3A	0.6186	0.4059	−0.2598	0.023*	
C4	0.64553 (14)	0.52522 (11)	−0.10278 (9)	0.01897 (18)	
H4A	0.6326	0.4457	−0.0753	0.023*	
C5	0.67176 (13)	0.66317 (11)	−0.03354 (8)	0.01655 (17)	
H5A	0.6777	0.6774	0.0410	0.020*	
C6	0.68979 (12)	0.78324 (10)	−0.07211 (7)	0.01388 (15)	
C7	0.71903 (12)	0.93282 (10)	−0.00369 (7)	0.01363 (15)	
C8	0.74228 (12)	1.13195 (10)	0.13166 (8)	0.01435 (15)	
C9	0.74225 (13)	1.23678 (11)	0.22774 (8)	0.01755 (17)	
H9A	0.7034	1.2126	0.2872	0.021*	
C10	0.80202 (14)	1.37822 (12)	0.23185 (9)	0.02025 (18)	
H10A	0.8038	1.4529	0.2958	0.024*	
C11	0.86028 (14)	1.41416 (11)	0.14373 (9)	0.02055 (19)	
H11A	0.9011	1.5122	0.1498	0.025*	
C12	0.85915 (13)	1.30930 (11)	0.04864 (8)	0.01792 (17)	
H12A	0.8982	1.3335	−0.0107	0.022*	
C13	0.79834 (12)	1.16605 (10)	0.04318 (8)	0.01454 (16)	
C14	0.62110 (12)	0.90630 (11)	0.17087 (7)	0.01496 (16)	
H14A	0.5363	0.8169	0.1275	0.018*	
H14B	0.5515	0.9667	0.2183	0.018*	
C15	0.77308 (12)	0.86831 (10)	0.23893 (7)	0.01369 (15)	
C16	0.74231 (12)	0.84165 (10)	0.33383 (7)	0.01303 (15)	
C17	0.88347 (12)	0.81141 (10)	0.39986 (7)	0.01459 (16)	
C18	1.05599 (13)	0.81031 (11)	0.37185 (8)	0.01720 (17)	
H18A	1.1524	0.7925	0.4173	0.021*	
C19	1.08576 (13)	0.83566 (11)	0.27609 (8)	0.01798 (17)	
H19A	1.2026	0.8336	0.2558	0.022*	
C20	0.94569 (12)	0.86386 (11)	0.21028 (8)	0.01622 (17)	
H20A	0.9675	0.8803	0.1451	0.019*	
C21	0.6815 (2)	0.47733 (14)	−0.42586 (10)	0.0353 (3)	
H21A	0.6958	0.4884	−0.4959	0.053*	
H21B	0.5768	0.4012	−0.4344	0.053*	
H21C	0.7923	0.4517	−0.3944	0.053*	
C22	0.97989 (14)	0.78394 (12)	0.57027 (8)	0.02013 (19)	
H22A	0.9313	0.7807	0.6352	0.030*	
H22B	1.0733	0.8706	0.5866	0.030*	
H22C	1.0342	0.6986	0.5441	0.030*	
O1W	0.50926 (11)	0.14161 (9)	0.43879 (6)	0.02107 (15)	
H2W1	0.433 (3)	0.158 (2)	0.3932 (17)	0.055 (6)*	
H1W1	0.515 (3)	0.052 (2)	0.4097 (15)	0.043 (5)*	

### Atomic displacement parameters (Å^2^)

**Table:**

	*U*^11^	*U*^22^	*U*^33^	*U*^12^	*U*^13^	*U*^23^
O1	0.0253 (3)	0.0151 (3)	0.0131 (3)	0.0034 (3)	0.0034 (2)	0.0061 (2)
O2	0.0147 (3)	0.0218 (3)	0.0125 (3)	0.0045 (2)	0.0046 (2)	0.0073 (3)
O3	0.0351 (4)	0.0180 (3)	0.0133 (3)	0.0056 (3)	0.0046 (3)	0.0028 (3)
O4	0.0175 (3)	0.0300 (4)	0.0152 (3)	0.0056 (3)	0.0030 (2)	0.0138 (3)
N1	0.0164 (3)	0.0157 (4)	0.0143 (3)	0.0041 (3)	0.0039 (3)	0.0066 (3)
N2	0.0152 (3)	0.0165 (4)	0.0123 (3)	0.0033 (3)	0.0033 (2)	0.0063 (3)
C1	0.0154 (3)	0.0157 (4)	0.0138 (4)	0.0040 (3)	0.0030 (3)	0.0066 (3)
C2	0.0196 (4)	0.0177 (4)	0.0135 (4)	0.0047 (3)	0.0033 (3)	0.0048 (3)
C3	0.0217 (4)	0.0151 (4)	0.0195 (5)	0.0035 (3)	0.0029 (3)	0.0046 (3)
C4	0.0207 (4)	0.0164 (4)	0.0219 (5)	0.0033 (3)	0.0032 (3)	0.0095 (4)
C5	0.0178 (4)	0.0183 (4)	0.0158 (4)	0.0036 (3)	0.0033 (3)	0.0085 (3)
C6	0.0138 (3)	0.0156 (4)	0.0134 (4)	0.0035 (3)	0.0025 (3)	0.0058 (3)
C7	0.0139 (3)	0.0161 (4)	0.0121 (4)	0.0038 (3)	0.0024 (3)	0.0056 (3)
C8	0.0138 (3)	0.0169 (4)	0.0134 (4)	0.0039 (3)	0.0018 (3)	0.0058 (3)
C9	0.0168 (4)	0.0212 (4)	0.0139 (4)	0.0047 (3)	0.0017 (3)	0.0039 (3)
C10	0.0207 (4)	0.0197 (5)	0.0184 (4)	0.0051 (3)	0.0018 (3)	0.0025 (4)
C11	0.0217 (4)	0.0168 (4)	0.0226 (5)	0.0042 (3)	0.0024 (3)	0.0053 (4)
C12	0.0189 (4)	0.0170 (4)	0.0196 (4)	0.0040 (3)	0.0039 (3)	0.0078 (3)
C13	0.0145 (3)	0.0165 (4)	0.0139 (4)	0.0039 (3)	0.0026 (3)	0.0062 (3)
C14	0.0142 (3)	0.0203 (4)	0.0131 (4)	0.0037 (3)	0.0035 (3)	0.0086 (3)
C15	0.0139 (3)	0.0165 (4)	0.0117 (4)	0.0034 (3)	0.0026 (3)	0.0054 (3)
C16	0.0129 (3)	0.0150 (4)	0.0115 (4)	0.0026 (3)	0.0026 (3)	0.0043 (3)
C17	0.0158 (3)	0.0165 (4)	0.0128 (4)	0.0030 (3)	0.0021 (3)	0.0067 (3)
C18	0.0153 (4)	0.0206 (4)	0.0182 (4)	0.0048 (3)	0.0022 (3)	0.0095 (4)
C19	0.0144 (4)	0.0230 (5)	0.0195 (4)	0.0053 (3)	0.0044 (3)	0.0097 (4)
C20	0.0148 (3)	0.0214 (4)	0.0148 (4)	0.0045 (3)	0.0045 (3)	0.0078 (3)
C21	0.0633 (9)	0.0230 (6)	0.0184 (5)	0.0126 (6)	0.0104 (5)	0.0008 (4)
C22	0.0206 (4)	0.0265 (5)	0.0146 (4)	0.0048 (3)	−0.0004 (3)	0.0095 (4)
O1W	0.0267 (4)	0.0235 (4)	0.0158 (3)	0.0093 (3)	0.0049 (3)	0.0076 (3)

### Geometric parameters (Å, °)

**Table:**

O1—C1	1.3581 (11)	C10—C11	1.4107 (16)
O1—H1	0.8400	C10—H10A	0.9500
O2—C16	1.3734 (11)	C11—C12	1.3837 (15)
O2—H2	0.8400	C11—H11A	0.9500
O3—C2	1.3697 (12)	C12—C13	1.4008 (14)
O3—C21	1.4229 (14)	C12—H12A	0.9500
O4—C17	1.3624 (11)	C14—C15	1.5183 (12)
O4—C22	1.4312 (12)	C14—H14A	0.9900
N1—C7	1.3379 (12)	C14—H14B	0.9900
N1—C13	1.3824 (13)	C15—C16	1.3920 (13)
N2—C7	1.3757 (12)	C15—C20	1.3976 (12)
N2—C8	1.3917 (12)	C16—C17	1.4066 (12)
N2—C14	1.4599 (12)	C17—C18	1.3913 (13)
C1—C2	1.4021 (14)	C18—C19	1.3959 (14)
C1—C6	1.4094 (13)	C18—H18A	0.9500
C2—C3	1.3860 (14)	C19—C20	1.3893 (13)
C3—C4	1.3961 (15)	C19—H19A	0.9500
C3—H3A	0.9500	C20—H20A	0.9500
C4—C5	1.3810 (15)	C21—H21A	0.9800
C4—H4A	0.9500	C21—H21B	0.9800
C5—C6	1.4108 (13)	C21—H21C	0.9800
C5—H5A	0.9500	C22—H22A	0.9800
C6—C7	1.4669 (13)	C22—H22B	0.9800
C8—C9	1.3945 (14)	C22—H22C	0.9800
C8—C13	1.4010 (13)	O1W—H2W1	0.84 (2)
C9—C10	1.3874 (15)	O1W—H1W1	0.87 (2)
C9—H9A	0.9500		
			
C1—O1—H1	109.5	C11—C12—H12A	121.2
C16—O2—H2	109.5	C13—C12—H12A	121.2
C2—O3—C21	116.18 (9)	N1—C13—C12	130.25 (9)
C17—O4—C22	116.88 (8)	N1—C13—C8	109.19 (8)
C7—N1—C13	106.48 (8)	C12—C13—C8	120.56 (9)
C7—N2—C8	106.87 (8)	N2—C14—C15	112.59 (7)
C7—N2—C14	130.73 (8)	N2—C14—H14A	109.1
C8—N2—C14	122.38 (8)	C15—C14—H14A	109.1
O1—C1—C2	116.37 (8)	N2—C14—H14B	109.1
O1—C1—C6	123.45 (9)	C15—C14—H14B	109.1
C2—C1—C6	120.18 (8)	H14A—C14—H14B	107.8
O3—C2—C3	125.03 (9)	C16—C15—C20	118.99 (8)
O3—C2—C1	114.20 (8)	C16—C15—C14	119.49 (8)
C3—C2—C1	120.76 (9)	C20—C15—C14	121.49 (8)
C2—C3—C4	119.20 (9)	O2—C16—C15	119.26 (8)
C2—C3—H3A	120.4	O2—C16—C17	120.41 (8)
C4—C3—H3A	120.4	C15—C16—C17	120.29 (8)
C5—C4—C3	120.85 (9)	O4—C17—C18	125.31 (8)
C5—C4—H4A	119.6	O4—C17—C16	114.37 (8)
C3—C4—H4A	119.6	C18—C17—C16	120.31 (8)
C4—C5—C6	120.82 (9)	C17—C18—C19	119.19 (8)
C4—C5—H5A	119.6	C17—C18—H18A	120.4
C6—C5—H5A	119.6	C19—C18—H18A	120.4
C1—C6—C5	118.16 (9)	C20—C19—C18	120.45 (9)
C1—C6—C7	117.77 (8)	C20—C19—H19A	119.8
C5—C6—C7	124.03 (8)	C18—C19—H19A	119.8
N1—C7—N2	111.35 (8)	C19—C20—C15	120.73 (9)
N1—C7—C6	120.54 (8)	C19—C20—H20A	119.6
N2—C7—C6	128.11 (8)	C15—C20—H20A	119.6
N2—C8—C9	131.51 (9)	O3—C21—H21A	109.5
N2—C8—C13	106.08 (8)	O3—C21—H21B	109.5
C9—C8—C13	122.40 (9)	H21A—C21—H21B	109.5
C10—C9—C8	116.42 (9)	O3—C21—H21C	109.5
C10—C9—H9A	121.8	H21A—C21—H21C	109.5
C8—C9—H9A	121.8	H21B—C21—H21C	109.5
C9—C10—C11	121.84 (10)	O4—C22—H22A	109.5
C9—C10—H10A	119.1	O4—C22—H22B	109.5
C11—C10—H10A	119.1	H22A—C22—H22B	109.5
C12—C11—C10	121.25 (10)	O4—C22—H22C	109.5
C12—C11—H11A	119.4	H22A—C22—H22C	109.5
C10—C11—H11A	119.4	H22B—C22—H22C	109.5
C11—C12—C13	117.52 (9)	H2W1—O1W—H1W1	103.0 (18)
			
C21—O3—C2—C3	−20.13 (15)	C8—C9—C10—C11	−0.19 (14)
C21—O3—C2—C1	160.96 (10)	C9—C10—C11—C12	0.43 (16)
O1—C1—C2—O3	−1.55 (12)	C10—C11—C12—C13	−0.13 (15)
C6—C1—C2—O3	177.80 (8)	C7—N1—C13—C12	179.37 (10)
O1—C1—C2—C3	179.48 (9)	C7—N1—C13—C8	−0.59 (10)
C6—C1—C2—C3	−1.16 (14)	C11—C12—C13—N1	179.66 (9)
O3—C2—C3—C4	−179.16 (9)	C11—C12—C13—C8	−0.39 (14)
C1—C2—C3—C4	−0.31 (15)	N2—C8—C13—N1	−0.56 (10)
C2—C3—C4—C5	0.61 (15)	C9—C8—C13—N1	−179.40 (8)
C3—C4—C5—C6	0.58 (14)	N2—C8—C13—C12	179.47 (8)
O1—C1—C6—C5	−178.40 (8)	C9—C8—C13—C12	0.64 (14)
C2—C1—C6—C5	2.28 (13)	C7—N2—C14—C15	91.13 (11)
O1—C1—C6—C7	−0.65 (13)	C8—N2—C14—C15	−87.41 (10)
C2—C1—C6—C7	−179.96 (8)	N2—C14—C15—C16	157.87 (9)
C4—C5—C6—C1	−2.01 (13)	N2—C14—C15—C20	−20.07 (13)
C4—C5—C6—C7	−179.61 (9)	C20—C15—C16—O2	178.18 (9)
C13—N1—C7—N2	1.57 (10)	C14—C15—C16—O2	0.19 (13)
C13—N1—C7—C6	−178.56 (8)	C20—C15—C16—C17	0.30 (14)
C8—N2—C7—N1	−1.94 (10)	C14—C15—C16—C17	−177.69 (9)
C14—N2—C7—N1	179.36 (8)	C22—O4—C17—C18	11.29 (15)
C8—N2—C7—C6	178.21 (8)	C22—O4—C17—C16	−167.85 (9)
C14—N2—C7—C6	−0.50 (15)	O2—C16—C17—O4	2.44 (13)
C1—C6—C7—N1	−15.16 (12)	C15—C16—C17—O4	−179.70 (9)
C5—C6—C7—N1	162.45 (9)	O2—C16—C17—C18	−176.74 (9)
C1—C6—C7—N2	164.69 (9)	C15—C16—C17—C18	1.11 (15)
C5—C6—C7—N2	−17.71 (14)	O4—C17—C18—C19	179.16 (10)
C7—N2—C8—C9	−179.84 (9)	C16—C17—C18—C19	−1.75 (15)
C14—N2—C8—C9	−1.00 (15)	C17—C18—C19—C20	0.99 (16)
C7—N2—C8—C13	1.47 (9)	C18—C19—C20—C15	0.42 (16)
C14—N2—C8—C13	−179.69 (8)	C16—C15—C20—C19	−1.06 (15)
N2—C8—C9—C10	−178.84 (9)	C14—C15—C20—C19	176.89 (9)
C13—C8—C9—C10	−0.33 (13)		

### Hydrogen-bond geometry (Å, °)

**Table:**

*D*—H···*A*	*D*—H	H···*A*	*D*···*A*	*D*—H···*A*
O1—H1···N1	0.84	1.80	2.5447 (12)	147
O1W—H2W1···O1^i^	0.84 (2)	2.23 (2)	3.0151 (11)	155 (2)
O2—H2···O4	0.84	2.21	2.6650 (11)	114
O2—H2···O1W^ii^	0.84	1.95	2.7401 (11)	155
O1W—H1W1···O2^iii^	0.87 (2)	2.04 (2)	2.8987 (12)	168.5 (19)
C21—H21B···O1W^iv^	0.98	2.58	3.2762 (16)	128
C22—H22A···O3^v^	0.98	2.54	3.2071 (14)	126
C22—H22B···Cg1^vi^	0.98	2.80	3.5497 (13)	133

Symmetry codes: (i) −*x*+1, −*y*+1, −*z*; (ii) −*x*+1, −*y*+1, −*z*+1; (iii) *x*, *y*−1, *z*; (iv) *x*, *y*, *z*−1; (v) *x*, *y*, *z*+1; (vi) −*x*+2, −*y*+2, −*z*+1.
